# Evaluation of a water phantom detector‐applicator setup for independent experimental verification of ^106^Ru ocular brachytherapy applicators

**DOI:** 10.1002/mp.70404

**Published:** 2026-04-05

**Authors:** Simon Dahlander, Michael Andrássy, Daniela Frömberg, Linda Persson, Åsa Carlsson Tedgren

**Affiliations:** ^1^ Department of Oncology Pathology Karolinska Institute Stockholm Sweden; ^2^ Department of Nuclear Medicine and Medical Physics Karolinska University Hospital Stockholm Sweden; ^3^ Eckert & Ziegler BEBIG GmbH Berlin Germany; ^4^ Swedish Radiation Safety Authority Stockholm Sweden; ^5^ Department of Health, Medicine and Caring Sciences Linköping University Linköping Sweden

**Keywords:** dosimetry, QA, Ru‐106 brachytherapy

## Abstract

**Background:**

**^106^** Ru ophthalmic brachytherapy (BT) applicators are used for treating ocular tumors. While manufacturer‐provided dosimetry data is commonly used for treatment planning, independent quality assurance (QA) measurements are crucial. However, there is a lack of dedicated equipment and standardized protocols for clinical verification of ^106^Ru depth‐dose distributions.

**Purpose:**

The BetaCheck‐106™ is a prototype compact water phantom detector‐applicator setup enabling high precision alignment of ^106^Ru applicators and detectors. We assess the setup's compatibility with three commercially available detectors (microSilicon, microSilicon X and microDiamond) and its performance in determining full 1D depth‐current curves with these detectors. In addition, data from clinical QA‐tests of 20 ^106^Ru applicators was used to estimate inhomogeneities in the applicator's ^106^Ru coating.

**Methods:**

Measurements were conducted on two ^106^Ru applicators with different activity levels, one had been in clinical service for one year and one was measured before clinical use. Dose rates were recorded at 1 mm intervals from 2 mm to 10 mm in water using the BetaCheck‐106™ setup with the three detectors. Measurement precision and detector response were analyzed. Separately, inter‐applicator variability was analyzed using the aforementioned measurements of 20 ^106^Ru applicators.

**Results:**

The BetaCheck‐106™ demonstrated exceptional setup reproducibility (0.23%), enabling precise depth‐resolved measurements. Both of the silicon diode detectors examined provided stable and reproducible measurements. The diamond detector performed reproducibly for the high‐activity applicator but exhibited depth‐dependent signal instability for the low‐activity source, likely due to the detector's lower sensitivity. Normalized depth‐signal curves for the three detectors all had similar shape. Analysis of measured current per activity of 20 ^106^Ru applicators revealed a depth dependent inhomogeneity effect decreasing with depth.

**Conclusions:**

The BetaCheck‐106™ provides practical high‐reproducibility positioning of detector and applicator for ^106^Ru applicator measurements in water. The silicon detectors successfully characterized both high and low activity applicators up to 10 mm water depth. The diamond detector proved viable for measurements of the applicator with activity 17.8–18.4 MBq, but it lost stability with depth when measuring the one‐year old 8.3 MBq activity applicator.

## 1 INTRODUCTION

Ophthalmic tumors can be treated with brachytherapy (BT) using ophthalmic BT applicators containing the beta emitter ^106^Ru/^106^Rh (referred to here as ^106^Ru for simplicity). The max energy of the ^106^Ru decay is 3.54 MeV, the mean energy is 1.41 MeV and the half‐life is 371.5 days ± 0.21 days.^1^ The ophthalmic applicators are 1 mm thick concave plaques made out of silver. The concave side is coated with a layer of ^106^Ru and sealed by a thin foil of silver. During treatment, the applicators are surgically attached to the eye, with a typical treatment time on the order of days. The applicators are available in different sizes and shapes from the manufacturer Eckert&Ziegler BEBIG GmbH, Berlin, Germany. The applicators are reusable, and one individual applicator is typically in clinical service for a period of one year.

Each ^106^Ru applicator is characterized by the manufacturer using a plastic scintillation detector calibrated against a transfer normal (a CCB‐type ^106^Ru applicator), whose central surface absorbed‐dose‐rate to water was determined using the primary standard at the University of Wisconsin Medical Radiation Research Center (UWMRRC),^2^ a dedicated convex windowless extrapolation chamber. The manufacturer's dosimetry data is provided with each applicator in the form of a certificate, detailing the absorbed‐dose‐rate to water along the central axis at depths ranging from 0.83 mm to 10 mm.

Acceptance tests on radiation sources are an important part of quality assurance (QA) in BT. Verification of the source manufacturer's dosimetric data by the clinical user is a requirement to assure correct dose delivery, and it is mandatory in many countries as well as advised by several guiding documents.^3^, ^4^, ^5^, ^6^

Despite the importance of dosimetric verification, there is a notable lack of dedicated equipment and standardized measurement protocols for clinical QA of ^106^Ru applicators, as highlighted by the AAPM Task Group No. 221^3^. A recent international survey^7^ revealed that 40% of clinics do not perform any independent verification measurements for ^106^Ru applicators, relying on unverified manufacturer‐provided data for treatment planning. Among clinics that do perform verification measurements, a variety of phantoms, detectors, and calibration approaches are used, some verifying the absorbed dose rate at the 2 mm reference depth, and others assessing the full 1D depth‐dose curve. The survey did not clarify whether these clinics were performing dosimetry in units of Gray (Gy) through traceable calibration factors or if they did relative measurements. The most common approach for treatment planning was the use of the manufacturer‐provided 1D depth‐dose rate curve.

Experimental measurement of the steep 1D depth‐dose rate curve is challenging. At the applicator reference depth (2 mm from the surface), dose gradients are about 25–30% per mm, demanding submillimeter detector‐applicator alignment, and detectors with thin active volumes to minimize volume‐averaging effects.

The equipment investigated in this article is a prototype of a water phantom detector‐applicator setup, designed to enable effective and accurate acceptance testing of ^106^Ru ocular applicators using direct‐reading detectors positioned in water. It was developed by the source manufacturer (Eckert&Ziegler BEBIG) under the name BetaCheck‐106™ to support users' independent QA of the dose rate of ^106^Ru applicators.

Measurements yielding values of absorbed dose in absolute units of Gy requires traceable detector calibration coefficients and is addressed in Dahlander et al (2026).^8^ That publication develops and validates a method for dosimetry traceable to external beam standards. The aim of the present study is twofold: first to evaluate the precision of the setup's detector‐applicator alignment when measuring the ocular applicators from 2 mm to 10 mm depth in water; and second, to investigate the performance of three types of commercially available detectors: two types of silicon diodes and one diamond detector. We will also report results from the use of the setup equipment for clinical QA over the time period 2021–2024, which includes measurements on 20 CCB applicators. These measurements were originally conducted as part of routine QA to verify the dosimetric performance of applicators before their clinical use, but they also provide a unique opportunity to investigate applicator‐to‐applicator variability. It is well known that slight manufacturing differences can lead to inhomogeneities in the distribution of ^106^Ru across applicators. A Monte Carlo (MC) study by Zaragoza et al. (2017)^9^ suggested that the dosimetric effects of such inhomogeneities are most pronounced near the applicator surface, and decrease with depth. However, to our knowledge, there has been no published experimental investigation of this phenomenon using measurement data from a large number of applicators of the same model assessed under identical conditions. Data on experimental measurements of multiple plaques are rare and has been requested in the literature.^10^

## 2 METHODS

### 2.1 The detector‐applicator setup

**FIGURE 1:**
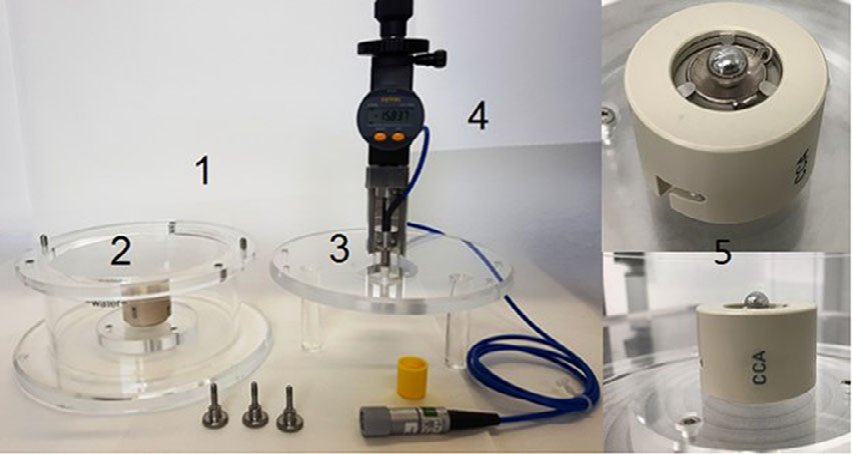
The BetaCheck‐106™ with components (1‐ phantom basin, 2‐ applicator holder, 3‐ detector attachment, 4‐ micrometer screw, 5‐ steel sphere).

The detector‐applicator setup is shown in Figure 1. It consists of a phantom basin (1) to be filled with water. The inner diameter of the basin (i.e. the diameter of the water volume) is 140 mm, and the height from phantom bottom the water fill mark is 53 mm. When filled to this mark, the water level is 29.5 mm above the applicator rim. For comparison, the manufacturer's water phantom is rectangular with inner dimensions 110 mm x 110 mm and a height from base to water mark of 51 mm, resulting in a water level 27.5 mm above the applicator rim. Given the limited range of the beta particles emitted by ^106^Ru, the dimensions of both phantoms are more than sufficient, and the difference in phantom size is not expected to affect measurements. Within the BetaCheck‐106™ phantom basin sits an exchangeable holder (2) which is specific to the applicator type and used to fixate the applicator position; this holder identical to the one used in the manufacturer's phantom. The lid of the phantom (3) has an attachment for the detector, which can be moved manually along the symmetry axis of the source using the high‐precision digital S246 micrometer screw (4), Sylvac, Yverdon, Switzerland. A reference‐class steel sphere (5) of diameter 8.000 mm accompanies the equipment and is used to calibrate the micrometer screw.

The setup procedure begins by fastening the applicator‐specific holder in the basin. The phantom is then filled with water, and the applicator is placed and securely locked into its holder. The detector is connected to a reference‐class electrometer, the electrometer is zeroed and leakage current is measured before the upper part of the device is positioned and fastened.

To calibrate the micrometer screw, the steel sphere is placed on the applicator and the detector is lowered using the micrometer screw until it stops at the sphere. At this point, the micrometer screw is zeroed, the detector is raised up, and the sphere is removed. The detector is now ready to be positioned at the desired depth in water. In this study, the vendor‐specified “Water equivalent window thickness” (see Table 1), was used as an offset to position the active volume of the detector at the intended depth relative to the applicator surface. In a continuation study (Dahlander et al 2026)^8^ MC simulated correction factors will be derived for the measurement scenario where the detector‐applicator is positioned in this way. These correction factors will effectively compensate for imperfections in this approximation of the effective point of measurement. These imperfections are less relevant in this study since the objective here is to evaluate the setup reproducibility and the detector repeatability.

**TABLE 1:** The diode detectors used in this study. The specifications were openly available from the manufacturer PTW.

	Diameter of sensitive volume (mm)	Thickness of sensitive volume (*µ*m)	Sensitive volume (mm^3^)	Water‐equivalent window thickness (mm)
microSilicon (60023, s/n 152002)	1.5	18	0.03	0.9
microSilicon X (60022, s/n 151950)	1.5	18	0.03	0.9
microDiamond (60019, s/n 122840)	2.2	1	0.004	1.0

Due to the detector's outer dimensions, measurements cannot be performed much closer than 2 mm from the applicator surface. This limitation was already anticipated in the past, leading to the selection of 2 mm rather than 1 mm as the source reference point in the international standard.^4^

### 2.2 Detectors, electrometer and ocular applicators

The detectors used in this study were the diode detectors microSilicon, microSilicon X, and microdiamond. The detector specifications can be found in Table 1; all detectors are from PTW GmbH, Freiburg, Germany. For brevity, the detectors will henceforth be referred to as *µ*Si, *µ*SiX and *µ*D, respectively. These are direct‐reading, waterproof detectors designed for small‐field external beam radiotherapy (EBRT) dosimetry, meaning they have active volumes small enough to allow for measurements in high dose gradients. This should make them suitable to measuring in the field of ^106^Ru eye applicators. It is recommended that for diode measurements of beta radiation from BT sources, the active volume of the detector should not exceed 1 mm in any dimension.^4^ The detectors used (see Table 1) do have diameters over 1 mm, but the major component of the dose gradient is in the depth direction, and the thickness of the detectors is much smaller than 1 mm. The two silicon detectors have the same specifications, but *µ*SiX is shielded to reduce low energy scatter and *µ*Si is not.

The electrometer used was the MAX 4000 Plus (s/n J190535), Standard Imaging, Madison, USA. The electrometer quality is reference‐class from 0.400 pA upwards, with a display resolution 0.001 pA.

Two ocular applicators from BEBIG with different activities were measured: CCB 3249, assessed before clinical use with an activity of 17.8–18.4 MBq at the time of measurement (variation in activity due to measurements made on different days), and CCB 3121, measured after approximately one year of clinical use, with an activity of 8.3 MBq during the measurements. The activities were taken from the applicator certificates.

### 2.3 Measurement procedure

Before the measurements, detectors were connected to the electrometer and pre‐irradiated with at least 2 Gy from the ^106^Ru applicator, the irradiation time required to reach 2 Gy was determined based on the certificate.

The phantom was filled with water within a 20–23°C temperature range (water left to stabilize at room temperature), no corrections for temperature were made.

After following the operational instructions overviewed in subsection 2A, the detector was moved towards the applicator to place the detector measurement point at the desired water equivalent distance from the center point of the applicator surface. One measurement cycle consisted of current measurements at all depths from 2 to 10 mm, performed as follows: Measurements were performed in 1 mm steps from 2 mm up to 10 mm. At each depth, 10 consecutive measurements were taken in a row without moving the detector. After completing the measurements at one depth, the detector was moved 1 mm to the next depth, and the process was repeated. Once one measurement cycle was completed, the procedure was repeated but with the depth direction reversed, starting at 10 mm and moving back down to 2 mm. Leakage current was determined as the mean of the current measured before and after the measurements without the source present. Current was determined by integrating over preset times determined on the electrometer. The charge collection time was varied depending on the strength of the current at the distance being measured, ranging from 10 sec at 2 mm depth up to 60 seconds at 10 mm depth.

For each detector, we performed four complete measurement cycles (as defined above: one cycle means measurements at each depth between 2–10 mm in 1 mm steps). These were conducted across two separate days, with two cycles per day, one cycle measuring 2–10 mm and one cycle measuring 10–2 mm. Each day's measurements used fully independent setups (including detector repositioning and system setup recalibration). This design allowed us to evaluate both intraday and inter‐day reproducibility.

### 2.4 Estimation of uncertainty

The analysis of uncertainty here follows the Joint Committee for Guides in Metrology (JCGM) Guide to the Expression of Uncertainty in Measurement.^11^ The different uncertainty components were assumed to be independent of each other.

To characterize the uncertainties evaluated by statistical analysis, we decompose the overall measured variability into two components:

**Setup reproducibility**: To evaluate the reproducibility of setting up the measurement, we focused on the *µ*Si detector, which has a high sensitivity and no shielding, meaning it should provide the strongest signal, and the high‐activity applicator (CCB 3249). The measurements at the 2 mm depth were used, where the dose gradient is steepest and the signal is highest, providing the most sensitive test of the setup's ability to reproduce measurement conditions.
**Measurement repeatability**: Within each individual measurement cycle, the standard deviation of the 10 repeated measurements at each depth captures the inherent random fluctuations of the collected charges (detector and electrometer). The average (or pooled) standard deviation from these individual cycles represents the measurement repeatability.

### 2.5 Estimating applicator inhomogeneity effects

In addition to the repeated measurements on the two applicators (CCB 3121 and CCB 3249) described in Section 2C, we have performed clinical QA measurements on 20 CCB applicators using the BetaCheck‐106™ setup since 2021. These QA measurements were performed in a similar manner to the procedure described in Section 2C, the differences being that only 1 measurement was taken per depth instead of 10, the electrometer used was the PTW Unidos Webline, the only detector used was the *µ*Si detector, and the measurements were not repeated on different days.

To estimate the effects of applicator inhomogeneity, the variability in measured signal current across the 20 applicators was analyzed. Since the applicators had different total activities, direct comparison of signals would be dominated by source‐strength variations. To eliminate this effect, the measured current at each depth was normalized by the total activity stated in the manufacturer's certificate for the respective applicator, yielding a signal of current per activity.

The observed relative standard deviation of these normalized values across the 20 applicators, denoted $u_{total}$, represents the observed variation in signal strength, independent of source strength, at every measured depth. This standard deviation arises from three contributions: true applicator‐to‐applicator variation due to inhomogeneity ($u_{applicator}$), the uncertainty of the activity measurement used for the normalization ($u_{activity}=2.3\%$), and the combined uncertainties for the *µ*Si detector measurements ($u_{measurement,\mu Si}$) as summarized in the uncertainty budget (Table 2). Assuming these contributions are uncorrelated, the variation due to applicator inhomogeneity was isolated by subtracting the other components in quadrature:

(1) $$\;u_{applicator}=\sqrt{u_{total}^{2}-u_{measurement,\mu Si}^{2}-u_{activity}^{2}}\;$$

where $u_{total},u_{measurement,\mu Si}$ and $u_{activity}$ are expressed as relative standard uncertainties. This approach allows us to estimate the component of dispersion in depth‐current measurements of different applicators which is due to applicator inhomogeneity.

**TABLE 2:** Uncertainty budget.

Uncertainty component	Type A (%)	Type B (%)
Half‐life of ^106^Ru		0.04
Half‐life resolution		0.05
Electrometer correction		0.1
Temperature correction		0.1
Recombination	–	–
Detector effective point of measurement position	–	–
Long term stability (microSilicon, microSilicon X, microdiamond)	0.1, 0.1, 0.2	
Measurement Repeatability (microSilicon, microSilicon X, microDiamond)	[0.26–0.76],[0.19–0.95],[0.22–2.51]	
Setup reproducibility	0.21	
Overall combined relative standard uncertainty(*k* = 1)	[0.39–0.81],[0.35–1.00],[0.41–2.54]	
Expanded relative measurement uncertainty (*k* = 2)	[0.78–1.62], [0.7–2], [0.82–5.08]	

## 3 RESULTS

### 3.1 Current strength

**FIGURE 2:**
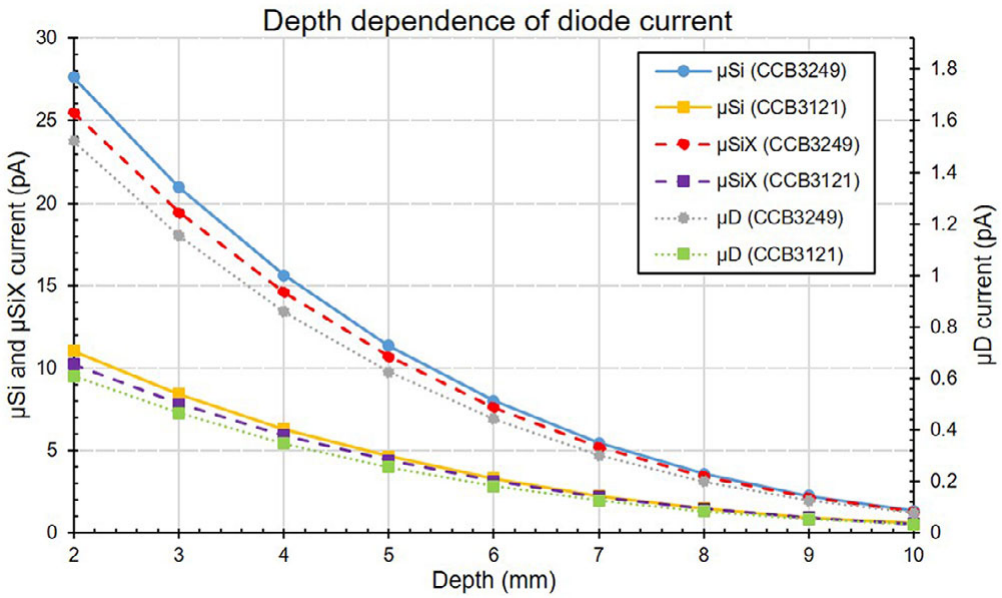
Current as a function of depth for the three detectors and two applicators. Note the different current‐scales for the silicon diodes (left *y*‐axis) and the diamond detector (right *y*‐axis). The size of the bars representing the expanded uncertainty (*k* = 2) at each point is comparable to the marker size, and hence not displayed.

Current as function of depth in water as measured by the three detectors is shown in Figure 2 (note that the silicon diodes and diamond detector use different current scales). For the applicator with higher activity (CCB3249), the *µ*Si current ranges from 28.6 pA at 2 mm to 1.37 pA at 10 mm, the *µ*SiX current ranges from 26.3 to 1.32 pA and the *µ*D current from 1.6 to 0.08 pA. For the applicator with lower activity (CCB3121), the *µ*Si current goes from 11.0 to 0.6 pA, the *µ*SiX from 10.2 to 0.6 pA and the *µ*D from 0.6 to 0.03 pA.

### 3.2 Measurement repeatability

**FIGURE 3:**
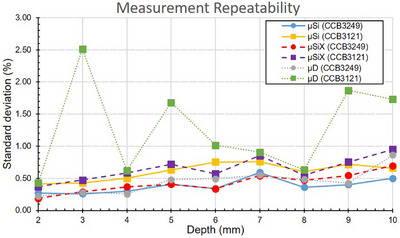
Relative standard deviation of 10 repeated measurements as a function of depth, representing random measurement repeatability within each setup. This reflects short‐term fluctuations due to detector noise, electrometer stability, and signal integration.

The contribution of measurement repeatability to standard deviation (as defined in section 2D) is shown in Figure 3. All detectors exhibited stable performance at the reference point (2 mm), with standard deviations below 1%. However, at greater depths, the *µ*D detector showed markedly increased variability for the lower‐activity applicator (CCB3121). The standard deviations from the *µ*D measurements for the older applicator fluctuated substantially, but for the newer applicator the *µ*D standard deviations remained below 1%. In contrast, the silicon diodes (*µ*Si and *µ*SiX) maintained high precision across the entire depth‐dose curve for both applicators, with standard deviations consistently remaining below 1%.

### 3.3 Setup reproducibility

The uncertainty of the micrometer screw was determined by repeatedly calibrating it on gauge blocks, yielding a standard deviation of 0.007 mm. Given the steep dose gradient (up to 30% per mm), this positional uncertainty corresponds to a signal uncertainty of:

(2) $$0.007\;\mathrm{mm}\;\times \;\frac{30\%}{\mathrm{mm}}=0.21\%$$

In addition, the observed standard deviation from repeated measurements at 2 mm with the *µ*Si detector was 0.10%. Combining these uncertainties in quadrature yields the total setup reproducibility:

(3) $$\;u_{setup}=\sqrt{{\left(0.10\%\;\right)}^{2}+{\left(0.21\%\right)}^{2}}\;\approx 0.23\%$$

This value represents the inherent reproducibility of the setup, incorporating both positional and detector‐specific contributions under optimal conditions.

### 3.4 Relative depth‐dose curves

**FIGURE 4:**
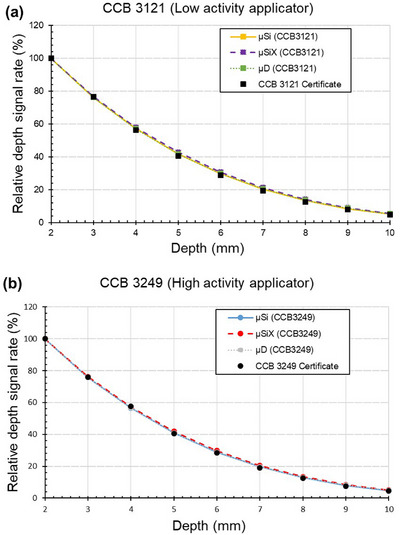
The relative depth signal curves (normalized to the 2 mm reference point) of the diodes and the manufacturer certificate data. In (a) the CCB 3121 applicator (measured after one year of clinical service) and in (b) the CCB 3249 applicator (measured before clinical service).

By normalizing measurements to the 2 mm reference point, the results from all three detectors could be compared with the dose rate values provided in the manufacturer's certificates, as shown in Figure 4.

### 3.5 Uncertainty in setup and current measurements

The quoted relative standard uncertainties are shown in Table 2. The overall combined relative standard uncertainty in the detector output was calculated for repeated measurements of one new (CCB 3249) and one old (CCB 3121) applicator, focusing on components directly derived from experimental reproducibility and literature‐based Type B uncertainties.

Key uncertainty components:

1. The half‐life of ^106^Ru was used for decay correction of measurements done on different days:
  - The half‐life of ^106^Ru is T_1/2 _= 371.5 days   ± 0.21 days,^1^ contributing a relative standard uncertainty of  $\frac{\ln\left(2\right)\cdot \Delta T_{1/2}}{T_{1/2}}=\frac{\ln(2)\cdot 0.21}{371.5}=0.04\%$.
  - Measurement time window: Measurements were assumed to have been conducted within a 12‐hour (0.5 day) period during the measurement days. The decay correction over this interval, assuming a rectangular probability distribution, introduces an uncertainty of   $\frac{1-e^{\frac{0.5}{371.5}}}{\sqrt{3}}=0.05\%$.
2. Electrometer calibration:
  - The relative uncertainty contributed by the stability of the MAX 4000 Plus electrometer was estimated to be 0.1%.
3. Temperature effects:
  - The water temperature during measurements ranged from 20°C to 23°C, and no temperature corrections were applied. The 0.1% uncertainty represents a maximum expected variation in detector response across this temperature range. This estimate is based on the temperature coefficients reported by Akino et al.,^12^, ^13^, ^14^ which demonstrated that the *µ*Si, *µ*SiX and *µ*D detectors exhibit minimal response variations of ≤0.1% per°C within this operational range. For the 3°C temperature interval in our study, the maximum cumulative variation was thus estimated to be 0.1%.
4. Recombination effects:
  - Assumed to be negligible. The stability in dose rate and DPP dependence reported by Akino et al.^12^, ^13^, ^14^ strongly suggests that recombination is negligible for the *µ*Si, *µ*SiX and *µ*D detectors in the context of ^106^Ru measurements.
5. Detector effective point of measurement position
  - Not relevant in this investigation, as we are not measuring in units of Gy.
6. Setup Reproducibility (Type A):
  - This uncertainty component accounts for variations introduced when repeating the entire measurement procedure, including detector repositioning, re‐zeroing of the electrometer, and recalibration of depth positioning. It is quantified as the standard deviation of mean dose rate values obtained from independent measurement setups on the same applicator.
7. Measurement Repeatability (Type A):
  - This component captures short‐term fluctuations due to detector noise, electrometer stability, and signal integration. It is calculated as the standard deviation of repeated dose rate measurements taken within the same setup. The pooled standard deviation across all depths provides an estimate of random measurement repeatability, depending on depth.
8. Detector long‐term stability (Type A):
  - Constancy checks of the *µ*Si and *µ*D detectors have been carried out in a ^60^Co field over three years. The *µ*SiX detector was assumed to have the same long‐term stability as the *µ*Si.

### 3.6 Inter‐applicator variation

**FIGURE 5:**
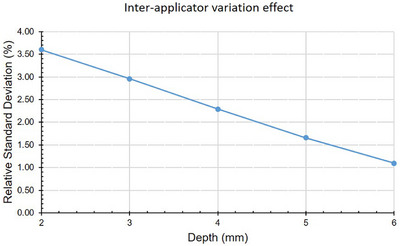
Relative standard deviation of current per activity measurements between 20 different CCB applicators.

The effects of applicator inhomogeneity (as defined by equation 1) is shown in Figure 5. For depths 7–10 mm, the calculated value of $u_{applicator}$ became non‐physical (i.e., the argument of the square root in equation (1) became negative). To our knowledge, this is the first study to report variability across 20 applicators of the same model, providing unique insights into manufacturing consistency.

## 4 DISCUSSION

### 4.1 Performance of the BetaCheck‐106™ setup

The inherent reproducibility of the BetaCheck‐106™ setup was 0.23%. The capability to easily position detectors reproducibly at steep dose gradients is particularly important, as small positional errors would otherwise dominate the measurement uncertainty. This excellent reproducibility allows for a clear and unbiased evaluation of detector performance, isolated from positional uncertainties.

### 4.2 Detector performance and suitability

All three detectors demonstrated stable measurements close to the applicator, the silicon diodes (*µ*Si and *µ*SiX) continued to be stable across the entire depth‐dose curve for both applicators. However, the lower sensitivity of the diamond detector (*µ*D) resulted in unreliable measurements at greater depths, as shown in Figure 3. For the higher activity applicator, at depths > 6 mm, the current from the *µ*D fell below 0.4 pA, which is outside the electrometer's effective range. However, the signal still appeared stable for the whole curve. For the low activity applicator, *µ*D fell below 0.4 pA at depths > 3 mm, and in this case the signal was not stable. It is unclear why the current falling below 0.4 pA gave rise to significant signal variations for the low activity applicator but not the higher activity one. Regardless, the signal instability due to the low currents indicates that the *µ*D might not be capable of accurately assessing applicators that have entered clinical service. The AAPM TG‐221 report^3^ specifies that if an applicator is suspected to have been damaged during clinical use, the medical physicist must repeat standard acceptance tests. It should be noted that these tests do not explicitly require dosimetric measurements. However, if a method for performing such measurements were readily available, one could argue that incorporating them into post‐damage QA checks would enhance the assessment of applicator integrity, ensuring continued dosimetric accuracy.

The normalized depth‐dose curves for the three detectors were mutually consistent. This internal agreement across detector types also aligns with the general shape of the depth‐dose distribution provided in the manufacturer's certificate data, as shown in Figure 4. Despite this overall consistency in curve shape, there are known detector‐specific limitations which needs to be discussed when evaluating detector performance.

Silicon‐based detectors like the *µ*Si and *µ*SiX exhibit energy dependence due to silicon's higher atomic number (*Z* = 14) compared to water (*Z* = 7.4). This affects beta particle interactions, leading to an over‐response at lower energies, which become more prevalent at greater depths as the beta spectrum softens. The mass collision stopping power ratio of water to silicon varies by up to 4% across the energy range 0.05–1.5 MeV,^15^ contributing to depth‐dependent uncertainties if not corrected for.

In contrast, the diamond detector (*µ*D), with near‐water equivalence (*Z* = 6), is expected to have a lower energy dependence,^16^ aligning more closely with absorbed dose to water. However, the *µ*D has a significantly lower sensitivity which limits its practical utility for full depth‐dose characterization, particularly for low‐activity applicators where signal‐to‐noise ratios deteriorate rapidly. A previous study^17^ performing measurements on CCB/CCA/CCX applicators up to 6 mm water depth found that deviations from manufacturer values up to 10% were detectable with the *µ*D detector.

The shielding on the *µ*SiX detector can be a disadvantage for non‐relative measurements. Since the parts of the radiation field that is screened out still delivers clinically relevant doses. The *µ*Si detector does not have this shielding, and its stability and sufficient sensitivity across the depth range makes it a pragmatic choice for clinical QA. However, its energy dependence introduces uncertainties, particularly at depths where beta energy degradation is significant. For dosimetry in units of Gy, this is something one has to correct for.

### 4.3 Applicator inhomogeneity

The analysis which aimed at isolating the component of variability on measured depth‐currents attributable to inhomogeneity in the ^106^Ru distribution for a batch of applicators shows a clear trend (see Figure 5) where the inhomogeneity effects decrease with depth. This result is physically intuitive, the effects of localized inhomogeneities on the applicator surface are expected to be averaged out with distance. At depths 7–10 mm, inhomogeneity effects could not be detected at all. However, this must be interpreted with caution. The present analysis is fundamentally limited by the relatively large uncertainty associated with the certificate activity values ($u_{activity}=2.3\%$), which propagates significantly into the normalized signal. A more rigorous quantification of the depth‐dependence of applicator inhomogeneity would require an activity measurement with a lower uncertainty. This would allow for a more precise deconvolution of inhomogeneity effects from other contributing factors. Nevertheless, these results suggest that manufacturing inhomogeneity effects are most pronounced near the applicator surface, in line with results by Zaragoza et al. (2017).^9^

### 4.4 Limitations of manufacturer‐dependent calibration methods

In an article by Ruiz et al. (2024),^18^ ^106^Ru applicators were measured using the BetaCheck‐106™ setup with a diode detector that had a calibration coefficient provided by Eckert & Ziegler BEBIG. While this approach provides a practical starting point for clinical QA, it creates a dependency on the manufacturer's calibration methodology. The manufacturer's plastic scintillator is calibrated using surface dose rate measurements from a single reference applicator, traceable to UWMRRC's extrapolation chamber.

Our observations of applicator‐to‐applicator variability (see Figure 5) demonstrate measureable differences between different applicators of the same model, likely arising from manufacturing variations in the ^106^Ru layer. The clinical significance of this variability and its potential impact on dosimetric accuracy warrants further investigation, particularly considering that the calibration approach is applied across applicator models beyond the CCB type used as the reference.

In addition, the significant changes in the ^106^Ru spectrum with depth pose an additional consideration. A calibration factor that is derived from surface measurements might not be able to account for depth‐dependent detector response variations. Together, these observations highlight the value of independent verification methods to complement manufacturer‐provided data.

### 4.5 Towards independent traceability and future directions

These considerations emphasize that, if complemented by a robust independent detector calibration method for measuring absorbed dose rate to water in absolute units of Gy, the BetaCheck‐106™ setup can serve as a reliable method for verifying dosimetry certificate data.

Currently, the National Physical Laboratory (NPL) is the only national metrology institute offering a calibration service for beta‐emitting ocular sources.^3^ This service utilizes 5 mm diameter by 0.5 mm thick alanine pellets arranged in stacks. This method involves measurements in a phantom made of WT1, a material which can be assumed to be water‐equivalent for EBRT fields. However, the assumption of water‐equivalence for WT1 and alanine in the ^106^Ru field has not been thoroughly validated, raising potential concerns about the accuracy of these measurements in the context of this source. In Dahlander et al (2026),^8^ through a collaboration with NPL, this water‐equivalence was evaluated using MC simulations.

In contrast, the BetaCheck‐106™ setup offers the advantage of performing measurements directly in water, the recommended reference medium. This eliminates the need for assumptions about the water‐equivalence of phantom materials, providing a more direct and potentially more accurate method for verifying dosimetric data. By measuring in water, the BetaCheck‐106™ setup aligns more closely with the conditions specified in dosimetry protocols, reducing uncertainties associated with material properties and enhancing the reliability of the results. However, the water‐equivalence of the detector itself in the ^106^Ru field requires further investigation.

Given the challenges associated with existing calibration routes, an alternate traceability chain would be to calibrate detectors against external beam standards for absorbed dose to water, following the approach by Lax (1991).^15^ This method would allow clinics to perform their own detector calibrations, which, combined with the BetaCheck‐106™ setup, would enable fully independent dosimetric measurements of ^106^Ru applicators. Such a method is presented in Dahlander et al (2026),^8^ where a traceable calibration protocol using microSilicon detectors and MC‐derived correction factors is presented.

## 5 CONCLUSION

The results of this study demonstrate that the BetaCheck‐106™ setup, equipped with appropriate detectors and a high precision electrometer, provides a robust method for verifying the depth‐dose curves of ^106^Ru ophthalmic applicators, as shown in Figure 4. The unshielded *µ*Si detector offered a balance between sensitivity and accuracy across the full depth‐dose curve. The shielded *µ*SiX diode was also a strong candidate, but its shielding might have slightly affected its response to the unique geometry of the ^106^Ru field. The *µ*D showed limitations in sensitivity, making it unsuitable for verification measurements of the full depth‐dose curve for low‐activity applicators. However, it performs reliably when measuring at the reference point and with high activity applicators.

Complemented by method(s) to calibrate the diode detector to measure absorbed dose rates to water in absolute units of Gy, the investigated detector‐phantom setup will fill an existing gap. The manufacturer's calibration method provides a practical starting point; in Dahlander et al. (2026),^8^ we present an alternative calibration methodology independent of the manufacturer.

In conclusion, the BetaCheck‐106™ setup, paired with an appropriate detector, represents a significant step forward in the dosimetry of ^106^Ru applicators. Future work should focus on developing and validating independent calibration methods to further enhance the accuracy and reliability of dosimetric verification. By addressing these challenges, this approach has the potential to empower clinical users with an effective and reliable method for acceptance tests of ^106^Ru ophthalmic applicators. Together with the calibration method described in Dahlander et al. (2026),^8^ this setup forms a complete methodology for acceptance testing and dosimetric verification of ^106^Ru applicators.

## CONFLICT OF INTEREST STATEMENT

M Andrassy has been, and D Frömberg is currently employed by Eckert & Ziegler BEBIG. S Dahlander, Å Carlsson Tedgren and L Persson have no conflict of interest to declare.

## DECLARATION OF GENERATIVE AI AND AI‐ASSISTED TECHNOLOGIES IN THE WRITING PROCESS

During the preparation of this work the authors used ChatGPT in order to make the language more fluent. After using this tool, the authors reviewed and edited the content as needed and take full responsibility for the content of the publication.
